# Therapeutic effects of lentinan on inflammatory bowel disease and colitis‐associated cancer

**DOI:** 10.1111/jcmm.13897

**Published:** 2018-11-24

**Authors:** Yanrong Liu, Jianmin Zhao, Yali Zhao, Shumin Zong, Yixuan Tian, Shuang Chen, Meng Li, Huijuan Liu, Qiang Zhang, Xueshuang Jing, Bo Sun, Hongzhi Wang, Tao Sun, Cheng Yang

**Affiliations:** ^1^ Tianjin Key Laboratory of Molecular Drug Research Tianjin International Joint Academy of Biomedicine Tianjin China; ^2^ Drug Safety Evaluation Center Tianjin International Joint Academy of Biomedicine Tianjin China; ^3^ Department of Pathology Hospital of Shun Yi District Beijing China; ^4^ State Key Laboratory of Medicinal Chemical Biology and College of Pharmacy Nankai University Tianjin China

**Keywords:** colitis‐associated cancer, inflammatory bowel disease, lentinan, TLR4

## Abstract

In this study, we investigated the therapeutic potential of lentinan in mouse models of inflammatory bowel disease (IBD) and colitis‐associated cancer (CAC). Lentinan decreased the disease activity index and macroscopic and microscopic colon tissue damage in dextran sulphate sodium (DSS)‐induced or TNBS‐induced models of colitis. High‐dose lentinan was more effective than salicylazosulfapyridine in the mouse models of colitis. Lentinan decreased the number of tumours, inflammatory cell infiltration, atypical hyperplasia and nuclear atypia in azoxymethane/DSS‐induced CAC model. It also decreased the expression of pro‐inflammatory cytokines, such as IL‐13 and CD30L, in IBD and CAC model mice possibly by inhibiting Toll‐like receptor 4 (TLR4)/NF‐κB signalling and the expression of colon cancer markers, such as carcinoembryonic antigen, cytokeratin 8, CK18 and p53, in CAC model mice. In addition, lentinan restored the intestinal bacterial microbiotal community structure in IBD model mice. Thus, it shows therapeutic potential in IBD and CAC model mice possibly by inhibiting TLR4/NF‐κB signalling‐mediated inflammatory responses and disruption of the intestinal microbiotal structure.

## 1 INTRODUCTION

Inflammatory bowel disease (IBD) is a disease that affects millions of people and includes ulcerative colitis (UC) and Crohn's disease (CD). IBD is a critical risk factor for colitis‐associated cancer (CAC), which was first recognized by Crohn and Rosenberg in 1925 and accounts for one‐sixth of all deaths in UC patients.^1^, ^2^, ^3^ However, currently available drugs to treat IBD and prevent CAC are not satisfactory. Hence, new and improved drugs are necessary.

IBD is influenced by genetic and environmental factors, infections and immune system disorders.^4^ TLRs are the key mediators of innate host defenses in the intestine that maintain mucosal immune homoeostasis. Toll‐like receptor 4 (TLR4) signalling induces the inflammatory response through the activation of nuclear factor kappa‐light‐chain‐enhancer of activated B cells (NF‐κB).^5^ Recent evidence shows that intestinal microbiota also plays a critical role in the pathogenesis of IBD.^6^ For centuries, Chinese culture has treasured mushrooms as a health food, an “elixir of life.”^7^ Mushrooms have been consumed worldwide to maintain general human health because of the general understanding that they have antioxidant, anticancer, antidiabetic, anti‐allergic, immunomodulating, cardioprotective, anticholesterolaemic, antiviral, antibacterial, antiparasitic, antifungal, detoxification and hepatoprotective effects; they also protect against inflammatory processes and tumour development.^8^, ^9^, ^10^, ^11^ The consumption of dietary mushrooms also decreases breast cancer risk in postmenopausal women.^12^ A number of bioactive compounds have been identified in mushroom species. The main constituents are polysaccharides.^13^ A hot‐water mycelial extract, called lentinan, has been widely used as a preventive and therapeutic agent for cancer patients in Japan and China.^14^, ^15^, ^16^ It is a polysaccharide with β‐1,3‐linked glucose backbone and β‐1,6‐linked glucose branches.^17^ Lentinan shows beneficial effects in DSS‐induced acute colitis mouse model because of its intestinal anti‐inflammatory activity.^18^ However, the therapeutic potential of lentinan in chronic UC, CD and CAC remains unknown. We have suggested that lentinan can exhibit therapeutic effects both on UC and CD and prevent CAC.

Therefore, we investigated the therapeutic efficacy of lentinan in chemically induced acute and chronic colitis and CAC mouse models.

## 2 MATERIALS AND METHODS

### 2.1 Reagents

Lentinan was obtained from Kaifeng Pharmaceutical Co. (Henan, China). The structure of lentinan is shown in Figure S1. Salicylazosulfapyridine (SASP; purity ≥98.0%), TNBS, azoxymethane (AOM) and LPS were purchased from Sigma‐Aldrich Co. (St. Louis, MO, USA). DSS was purchased from MP Biomedicals (Santa Ana, CA, USA, molecular weight; 36 000‐50 000).

### 2.2 Experimental animals and chemically induced mouse models of colitis

**Figure 1:**
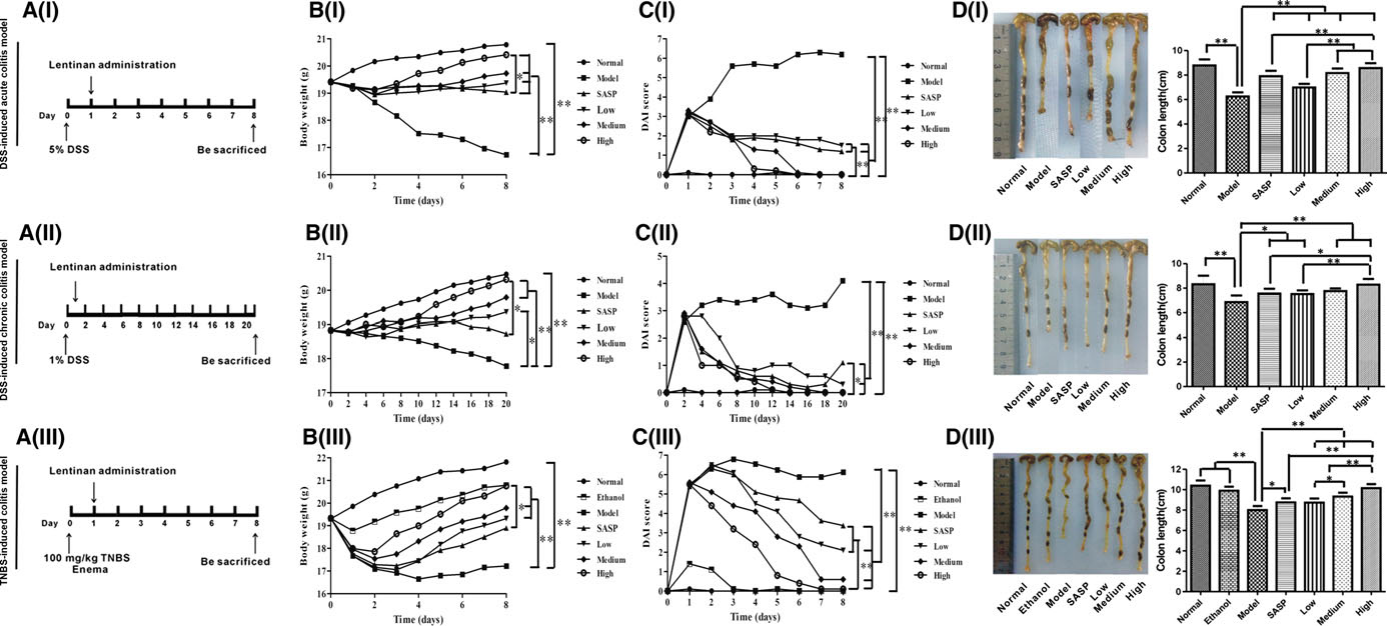
Effects of lentinan treatment on body weight, disease activity index (DAI), and colon length in DSS‐induced and TNBS‐induced mouse models of colitis. A, Schematic representation showing the establishment of mouse models of DSS‐induced acute or chronic colitis and TNBS‐induced colitis and lentinan administration in (i) acute colitis, (ii) chronic colitis and (iii) TNBS‐induced colitis model mice. B, Body weight changes in low‐, medium‐, and high‐dose lentinan‐treated (i) acute colitis, (ii) chronic colitis and (iii) TNBS‐induced colitis model mice. C, DAI in low‐, medium‐, and high‐dose lentinan‐treated (i) acute colitis, (ii) chronic colitis and (iii) TNBS‐induced colitis model mice. D, Colon lengths in low‐, medium‐, and high‐dose lentinan‐treated (i) acute colitis, (ii) chronic colitis and (iii) TNBS‐induced colitis model mice. *denotes *P *<* *0.05; **denotes *P *<* *0.01

Female C57BL/6 or BALB/c mice (18‐22 g) were purchased from the Animal Center Academy of Military Medical Science (Beijing, China) and bred under specific pathogen‐free conditions. The experiments were performed according to a protocol approved by the Animal Ethics Committee of the Tianjin International Joint Academy of Biotechnology and Medicine. The mice were allowed to acclimatize for 7 days before being used in any experiments. Different DSS concentrations and administration cycles induce acute or chronic colitis model in mice.^19^, ^20^, ^21^ Rectal administration of TNBS induces pathology similar to human CD.^22^ Mice were divided into the following six groups (n = 10/group) for the DSS‐induced mouse model: normal control (Normal); model control (Model); SASP (Sulfasalazine); and low‐, medium‐ and high‐dose lentinan (5, 10 and 20 mg/kg, respectively) groups. For the TNBS‐induced colitis model,^19^, ^20^ an additional solvent control group (Ethanol) was added in addition to the above groups. DSS‐induced acute colitis was induced in C57BL/6 mice by oral administration of 5% DSS in drinking water for 8 days. DSS‐induced chronic colitis was induced in C57BL/6 mice by administering 1% DSS orally for 21 days according to previously published protocols. Fresh DSS solution was prepared every day. Normal control mice were given drinking water only. TNBS‐induced colitis model was induced in BALB/c mice by administering an enema of 120 mg/kg TNBS in 50% ethanol.^22^, ^23^ The normal control and solvent control groups were administered with enemas of normal saline and 50% ethanol respectively. A schematic overview of the animal model established in this study and the lentinan administration methods used are shown in Figure 1A. C57BL/6 mice were used for DSS‐induced colitis because cryptitis and crypt abscesses can be found in half of the DSS‐treated C57BL/6JOlaHsd mice, while in BALB/cAnNHsd mice, cryptitis was rarely observed. Reepithelization of the distal part of the colon can be found as early as day 5 of DSS treatment in C57BL/6JOlaHsd but not in BALB/cAnNHsd mice.^24^ Moreover, BALB/c mice were used for induction of colitis by TNBS because BALB/c strains are susceptible to TNBS induction, while C57BL6 are resistant.^20^, ^25^

### 2.3 Colitis model and lentinan treatment

The long‐term retention test of lentinan showed that lentinan was stable under the conditions of temperature (23‐27°C) and relative humidity (50%‐70%) for 12 months. The study of tissue distribution of lentinan in mice after lentinan intraperitoneal injection showed that lentinan was mainly distributed in the spleen and liver and less distributed in the heart, lungs and kidneys (the above data were provided by the manufacturer's instruction). Inflammation and injury in the colons of the model mice were documented approximately 24 hours after oral DSS or TNBS enema administration.^26^, ^27^ Lentinan was dissolved in double‐distilled water (ddH_2_O) and then was administered to the model mice at 24 hours after DSS or TNBS administration. Normal and DSS‐only model mice were administered with ddH_2_O intragastrically once per day. The DSS‐induced acute colitis model or TNBS‐induced colitis model mice were administered with lentinan solution (low dose, 5 mg/kg; medium dose, 10 mg/kg; and high dose, 20 mg/kg) intragastrically, once per day for 7 days. The DSS‐ induced chronic colitis model mice were administered with the same amount of lentinan solution for 20 days. SASP was used as the positive drug control (at a dose of 200 mg/kg) because it is widely used as an anti‐colitis agent in human IBD therapy and serves as a positive control in studies of experimental colitis models.^28^, ^29^ It is indicated in the treatment of mild‐to‐moderate UC and as adjunctive treatment of severe UC.^30^

The C57BL/6 mice of the model and normal control groups were pretreated with 20 mg/kg lentinan and ddH_2_O daily for 7 days. Then, the model group mice were administered with 5% DSS in drinking water for 7 days, whereas the normal control mice were administered with normal drinking water only. The mice were monitored for body weight, stool consistency and bloody stool every day until they were killed. The colitis assessments were performed as described in the Data S1.

### 2.4 CAC induction and lentinan treatment

BALB/c mice (n = 30) were randomly assigned to the normal, model and lentinan‐treatment groups. The CAC animal model was established by intraperitoneal injection of 12 mg/kg AOM in physiological saline. Five days after AOM injection, the mice were provided with 2% DSS in drinking water for seven consecutive days, followed by a 14‐day recovery period. This cycle of induction was repeated thrice to establish the CAC animal model.^31^ The mice of the normal control group were treated only with physiological saline instead of AOM and DSS. Lentinan (20 mg/kg) was administered daily starting at the beginning of the third cycle and was continued until the mice were killed. The number of colonic tumours was subsequently counted, and a small piece of the colon was used for haematoxylin‐eosin (H&E)‐stained sections and immunohistochemical staining with colon cancer markers such as p53, carcinoembryonic antigen (CEA), cytokeratin 8 (CK8) and CK18. Moreover, the expression of cytokines was also investigated.

### 2.5 RAW264.7 cell culture, TLR4 overexpression, and lentinan treatment

Mouse macrophage RAW264.7 cells were purchased from Keygen Biotech (Nanjing, China) and authenticated by short tandem repeat analysis. They were cultured in RPMI‐1640 medium supplemented with 10% foetal bovine serum, 100 units/mL penicillin and 100 mg/mL streptomycin at 37°C and 5% CO_2_.

The mammalian expression vector encoding the mouse TLR4 OR, pReceiver‐B02‐TLR4 (GeneCopoeia, Germantown, MD, USA) was transfected into RAW 264.7 cells using Lipofectamine 2000 (Invitrogen, USA). After 48 hours, the cells were treated with 1 μg/mL LPS in combination with different lentinan concentrations (0.5, 1, and 2 mg/mL) for 24 hours and analysed by Western blot.

### 2.6 Western blot detection

Detailed information is provided in the Data S1.

### 2.7 Calcium flux detection

RAW 264.7 cells (10^4^ cells/well) were seeded in a 384‐well plate and grown overnight. Then, the cells were incubated for 2 hours with 25 μL dye‐loading solution at 37°C (Molecular Devices, LLC, Sunnyvale, CA, USA). Then, the cells were stimulated by 1 μg/mL LPS for 1 hour followed by lentinan. Calcium flux was estimated by recording the fluorescence intensity in the Flex Station (Molecular Devices).

### 2.8 Immunofluorescence analyses

Detailed information is provided in the Data S1.

### 2.9 Immunohistochemical analyses

Detailed information is provided in the Data S1.

### 2.10 Dual‐luciferase reporter assays

Detailed information is provided in the Data S1.

### 2.11 Quantitative analysis of 40 mouse cytokines

RAW 264.7 cells were stimulated with 1 μg/mL LPS in combination with 1 mg/mL lentinan for 24 hours. The cell culture supernatants were collected and analysed for 40 different cytokines by cytokine antibody arrays (RayBiotech Inc., Georgia, USA).

### 2.12 16S rRNA sequencing of intestinal microbiota

The detailed protocol is shown in the Data S1.

### 2.13 Statistical analysis

Data were analysed using spss 17.0 (SPSS, Inc., Chicago, IL, USA)and expressed as the mean ± SD. Differences between groups were analysed by the Mann‐Whitney *U* test and one‐way analysis of variance. *P *<* *0.05 was considered statistically significant.

## 3 RESULTS

### 3.1 Lentinan treatment is more effective than SASP in the chemically induced mouse models of colitis

We tested the effects of lentinan on IBD progression in mouse models of colitis. In the model control group, we observed IBD‐like colitis, diarrhoea and bloody mucopurulent stools. The average body weight of the model group mice started to decline a day after DSS or TNBS administration. However, we observed that lentinan treatment prevented body weight loss in a concentration‐dependent manner when administered 24 hours after DSS and TNBS (Figure 1B). For example, the high‐dose lentinan group (20 mg/kg) restored body weight better than the SASP treatment group in the DSS‐induced acute colitis model (20.41 ± 0.67 g vs 19.04 ± 0.23 g, *P *=* *0.029; Figure 1B). The disease activity indexes (DAIs) of both lentinan‐treated and SASP groups were lower than that of the model group (Figure 1C). Moreover, the medium‐ and high‐dose lentinan‐treated groups had lower DAIs than the SASP group (Figure 1C). Furthermore, lentinan and SASP treatments prevented colon length shortening. The colon length was longer in the high‐dose lentinan group than in other groups, indicating that high‐dose lentinan was more effective in maintaining colon length than SASP in the DSS‐induced acute colitis model (8.57 ± 0.40 cm vs 7.91 ± 0.43 cm, *P *=* *0.002; Figure 1D). These results demonstrate that lentinan attenuates colitis more effectively than SASP.

### 3.2 Lentinan alleviates colonic damage in the IBD mouse model

**Figure 2:**
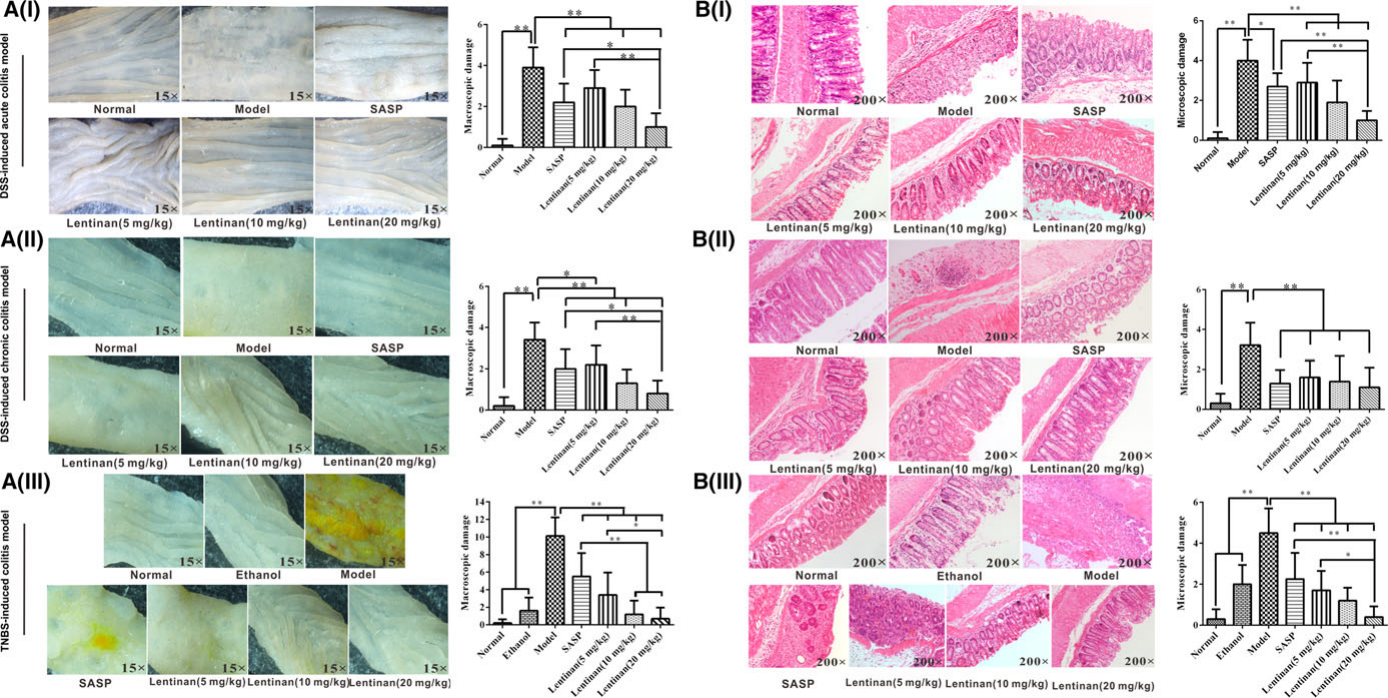
Histopathological analysis of mouse models of colitis after treatment with lentinan. A, Left panel shows representative photographs of the colons from normal, DSS‐induced acute, and chronic colitis and TNBS‐induced colitis mice treated with or without SASP and low‐, medium‐, and high‐dose lentinan. The right panel shows the macroscopic damage scores based on visual inspection of the histopathology. As shown, lentinan decreases colon damage scores in a concentration‐dependent manner. B, Left panel shows representative photographs of H&E‐stained colon pathological sections from normal, acute and chronic colitis and TNBS‐induced colitis mice treated with or without SASP and low‐, medium‐, and high‐dose lentinan. The inflammatory cell infiltration and crypt erosion are marked with blue or yellow arrows respectively. The foci of inflammatory cells in the model group are enlarged in the upper left corner. The right panel shows the microscopic damage scores based on the analysis of H&E‐stained pathological sections. Lentinan ameliorates colitis‐associated microscopic damage in the mouse model. *denotes *P *<* *0.05; **denotes *P *<* *0.01

We observed the disappearance of mucosal folds, visible erosion, ulcers, mucosal hyperaemia and oedema in the colon tissues of DSS/TNBS‐induced IBD model mice (Figure 2A,B). The colon tissues of DSS‐induced acute colitis model mice showed large amounts of mucus or purulent secretions in the enteric cavity. DSS‐induced chronic colitis model mice showed granulated intestinal mucosa with superficial ulcer formation, local mucosal hyperaemia and oedema. The colons of TNBS‐induced colitis model mice showed segmental ulcers; thicker intestinal walls; and the appearance of necrotic tissue, bowel perforations or megacolon. Lentinan decreased colon macroscopic damage scores more effectively than SASP (acute colitis model; 1.00 ± 0.67 vs 2.20 ± 0.92, *P *=* *0.004; Figure 2A). Histological sections of the colorectal tissues of the DSS‐induced acute and chronic colitis mouse models also revealed severe inflammatory lesions, crypt erosion and immune cell infiltration (neutrophils in acute colitis and lymphocytes in chronic colitis mouse models). Granuloma formation was observed in the colorectal tissues of the TNBS‐induced colitis mouse model. We observed smaller areas of erosion and reduced lymphocyte infiltration in lentinan‐treated colitis model mice and lower microscopic scores of high‐dose lentinan treament than SASP treament (Figure 2B). This result suggested that lentinan decreased colitis severity more effectively than SASP.

### 3.3 Lentinan pre‐treatment prevents DSS‐induced mouse models of colitis

**Figure 3:**
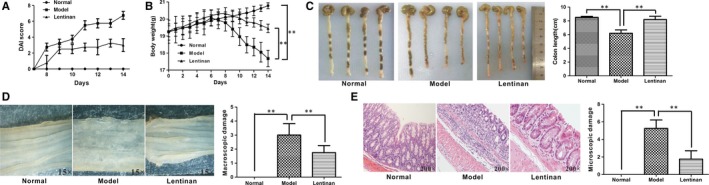
Lentinan pre‐treatment prevents DSS‐induced colitis. A, DAIs of normal, untreated and lentinan pre‐treated colitis model mice. B, Body weights of normal, untreated and lentinan pre‐treated colitis model mice. C, Colon lengths of normal, untreated and lentinan pre‐treated colitis model mice. D, Representative photographs of colons from normal, untreated and lentinan pre‐treated model mice and their macroscopic damage scores. E, Representative photographs of H&E‐stained colon sections from normal, untreated and lentinan pre‐treated model mice and their colon histological scores

We also investigated if lentinan prevented DSS‐induced mouse models of colitis by treating a group of mice with lentinan (20 mg/kg) before DSS induction. The lentinan pre‐treated mice showed lower DAIs (3.000 ± 0.82 vs 6.750 ± 0.50 reduced by nearly 50%, *P *=* *0.002, Figure 3A), less body weight loss (19.458 ± 0.25 g vs 17.685 ± 0.48 g *P *=* *0.005; Figure 3B) and longer colon lengths (8.200 ± 0.48 cm vs 6.200 ± 0.48 cm, *P *=* *0.001, Figure 3C) than untreated mice after DSS induction. Visual inspection of the colons showed that lentinan pre‐treatment inhibited ulcer formation leading to lower macroscopic damage scores than the model group (1.75 ± 0.50 vs 3.00 ± 0.81, *P *=* *0.040, Figure 3D). In addition, the histological scores of lentinan pretreated mice were lower than those of the model group mice (1.75 ± 0.96 vs 5.25 ± 0.96, *P *=* *0.002; Figure 3E). Lentinan pre‐treated mice also showed more intact mucous epithelium and lower neutrophil infiltration than untreated mice (Figure 3E).

### 3.4 Lentinan inhibits CAC development and progression

**Figure 4:**
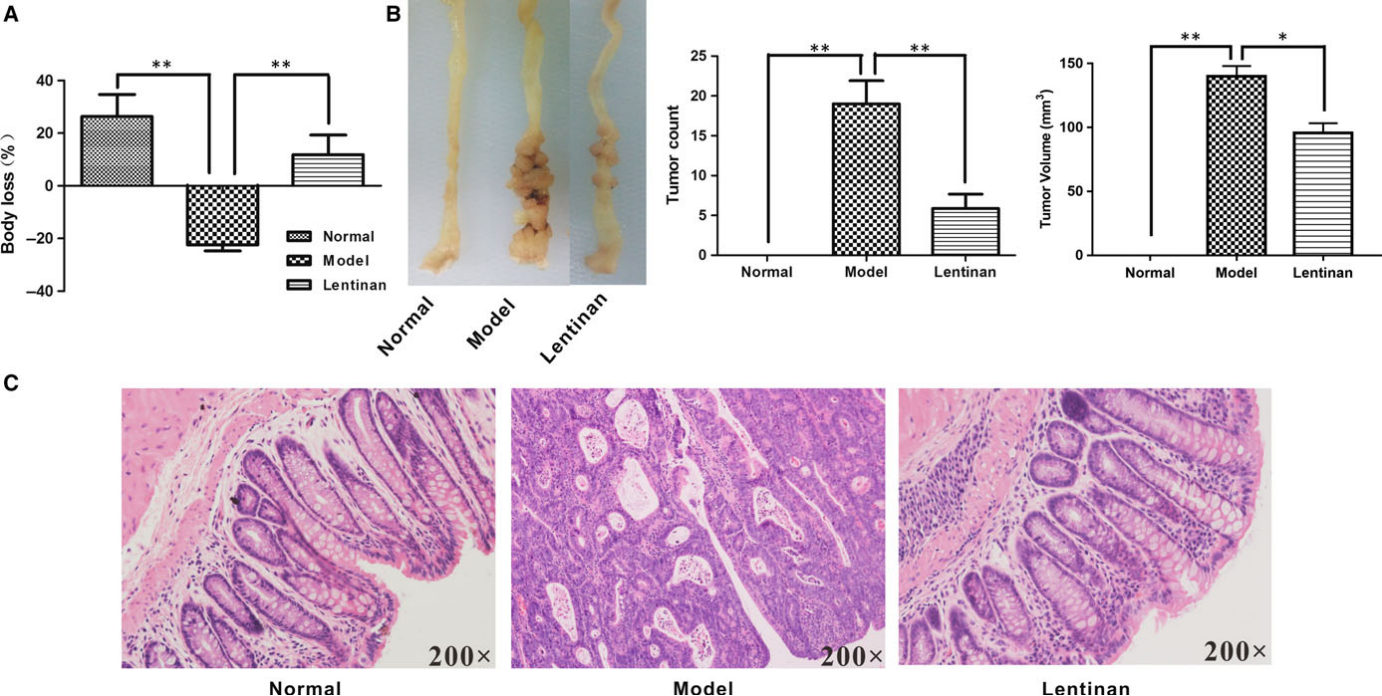
Lentinan treatment prevents CAC. A, Body weights of normal, untreated and lentinan pre‐treated CAC model mice. B, Representative photographs and data of colons from normal, untreated and lentinan pre‐treated CAC model mice showing tumor counts and volume. C, Representative photographs of H&E‐stained pathological sections of colons from normal, untreated and lentinan pre‐treated CAC model mice. 15× and 200× represent magnification of photographs; *denotes *P *<* *0.05; ** denotes *P *<* *0.01

Next, we investigated if lentinan inhibited colitis‐induced carcinogenesis in the AOM/DSS‐treated CAC mouse models. As shown in Figure 4A, AOM/DSS administration decreased the body weight, but lentinan‐treated mice showed higher body weight. Lentinan treatment decreased the number and size of tumours (Figure 4B), inflammatory cell infiltration, atypical hyperplasia and nuclear atypia than in model colon tissues (Figure 4C). This finding suggested that lentinan inhibited CAC development and progression.

### 3.5 Lentinan inhibits TLR4‐induced inflammation in vitro

**Figure 5:**
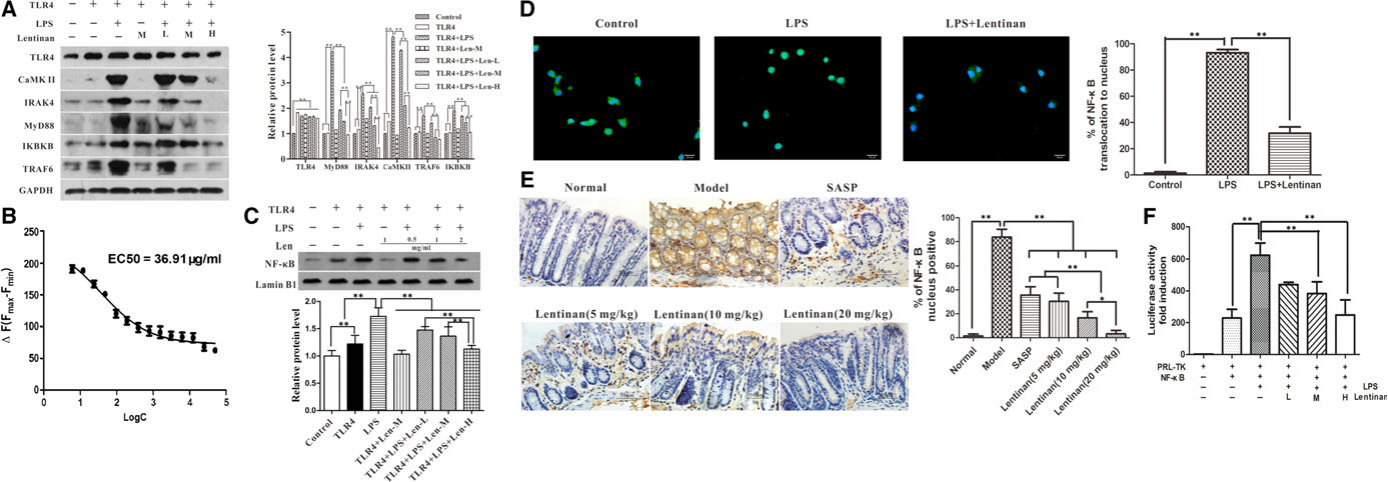
Lentinan inhibits TLR4 signalling pathway. A, Expression of TLR4 signalling pathway proteins including CaMKII, IRAK4, MyD88, IKBKB and TRAF6 in LPS‐stimulated RAW264.7 cells treated with or without 0.5, 1 and 2 mg/mL lentinan. B, FACS analysis of calcium influx in LPS‐stimulated RAW264.7 cells treated with or without 0.5, 1 and 2 mg/mL lentinan. The calcium influx signal decreases in a dose‐dependent manner upon lentinan treatment. C, Representative Western blot showing expression of NF‐κB p65 subunit in the nuclear extract from LPS‐stimulated RAW264.7 cells treated with or without 0.5, 1 and 2 mg/mL lentinan. D, Immunofluorescence micrographs showing the status of nuclear translocation of NF‐κB‐p65 in LPS‐stimulated RAW264.7 cells treated with or without 0.5, 1 and 2 mg/mL lentinan. E, Immunohistochemical staining images showing NF‐κB‐p65 levels in the colon from acute colitis model mice with or without lentinan treatment. F, Dual luciferase reporter assay analysis showing changes in NF‐κB activity in the presence and absence of lentinan. All the above data are represented as mean ± SD. *denotes *P *<* *0.05; **denotes *P *<* *0.01

Mucosal immune disorders lead to IBD as a result of TLR4 activation results in NF‐κB activation and cytokine expression in macrophages.^32^ We investigated if lentinan inhibited lipopolysaccharide (LPS)‐stimulated TLR4 signalling and reduced IBD progression. As shown in Figure 5A, lentinan inhibited LPS‐induced TLR4 signalling in TLR4‐overexpressing RAW264.7 cells stimulated by LPS. It reduced the expression of many downstream signalling key proteins such as calmodulin‐dependent protein kinase II (CaMKII); interleukin‐1 receptor‐associated kinase 4 (IRAK4); myeloid differentiation primary response gene 88 (MyD88); Inhibitor of kappa light polypeptide gene enhancer in B‐cells, kinase beta (IKBKB); and tumour necrosis factor receptor‐associated factor 6 (TRAF6).

LPS‐induced TLR4 activation increases calcium influx followed by CaMKII activation.^33^ We observed that lentinan treatment decreased calcium influx in a concentration‐dependent manner (Figure 5B). This finding suggested that lentinan decreased TLR4 signalling. Furthermore, Western blot, immunofluorescence and immunohistochemistry experiments showed that lentinan reduced the translocation of NF‐κB‐p65 to the nucleus (Figure 5C‐E). Dual‐luciferase reporter gene assay also demonstrated that lentinan reduced NF‐κB activity (Figure 5F). We also tested the efficacy of lentinan treatment in another colon epithelial carcinoma cell line, Caco‐2, and found that lentinan inhibited TLR4 pathway and nuclear translocation of NF‐κB (Figure S2).

### 3.6 Lentinan decreases the levels of IL‐13 and CD30L in LPS‐induced inflammation in vitro

**Figure 6:**
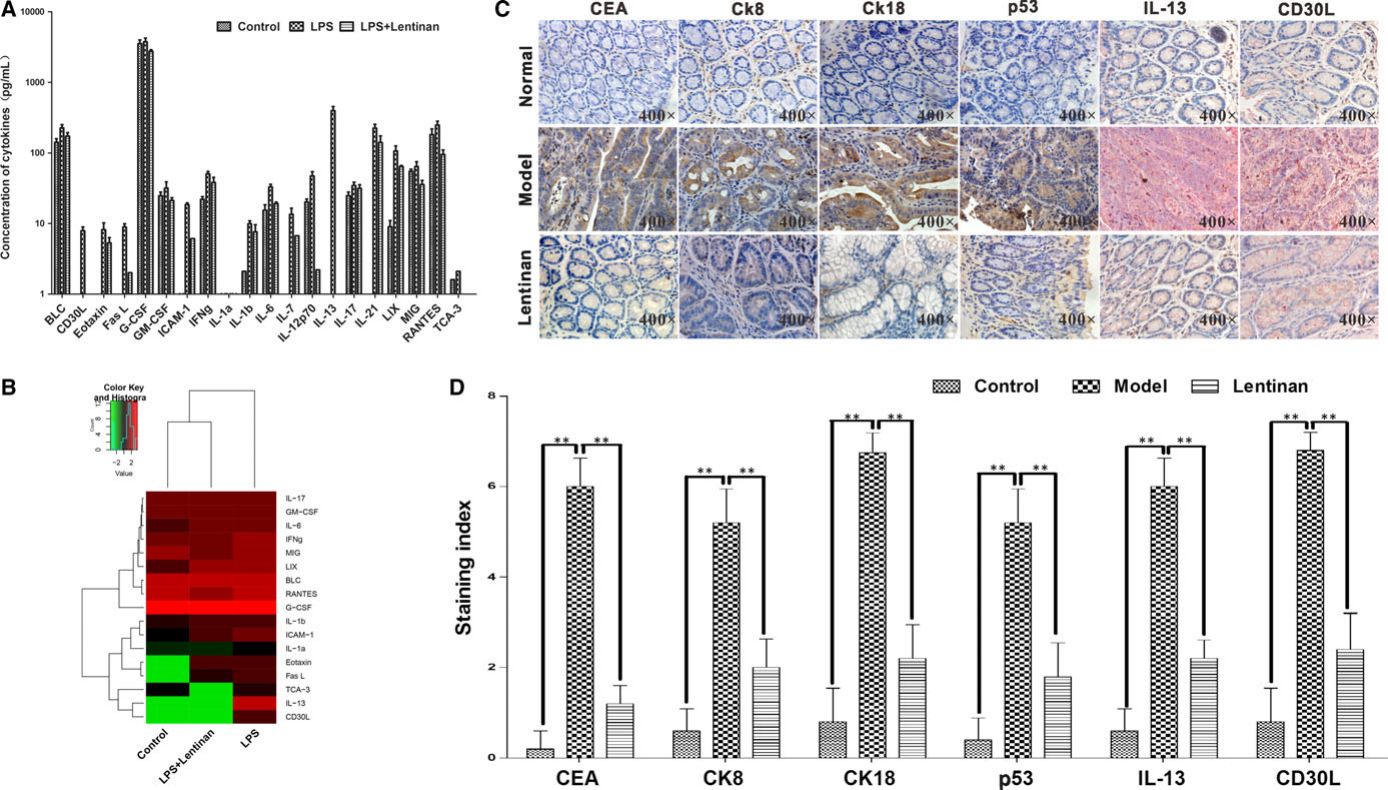
Lentinan inhibits the expression of inflammatory cytokines and colon cancer markers. A, Cytokine antibody chip assay analysis of the concentrations of cytokines from RAW264.7 cells treated with 1 mg/mL LPS in the presence or absence of 1 μg/mL lentinan, detected by cytokine antibody chip assay. B, Grouping analysis of the cytokine antibody chip assay results. C, Representative images of IHC staining of the cancer markers CEA, CK8, CK18 and p53 (all in brown) and the cytokines IL‐13 and CD30L (both in red) in colon tissues from CAC model mice treated with or without lentinan. The nuclear staining is coloured blue. D, Quantitative analysis of cancer markers and inflammatory cytokines based on the IHC staining in 5C. Data are represented as mean ± SD. *denotes *P *<* *0.05; **denotes *P *<* *0.01

The inflammatory cytokine antibody assay showed that lentinan also decreased the expression of the downstream inflammatory cytokines regulated by NF‐κB. As shown in Figure 6A, LPS induced 40 different types of inflammatory cytokines in RAW 264.7 macrophages, but lentinan treatment reduced the levels of these LPS‐induced inflammatory cytokines. Most significantly, 1 mg/mL of lentinan decreased the expression of IL‐13 and CD30L, which are critical inflammatory factors (Figure 6A,B). Clustering analysis demonstrated that the inflammatory cytokines in lentinan‐treated RAW 264.7 cells stimulated by LPS were more similar to control cells without LPS stimulation (Figure 6B).

### 3.7 Lentinan reduces the expression of IL‐13 and CD30L and colon cancer markers in CAC mouse models

Immunohistochemical analysis of colorectal tissues from the CAC mouse models showed that lentinan decreased the expression of IL‐13 and CD30L as well as the colon cancer markers such as CEA, CK8, CK18 and p53 (Figure 6C,D).

### 3.8 Lentinan restores the intestinal floral structure

**Figure 7:**
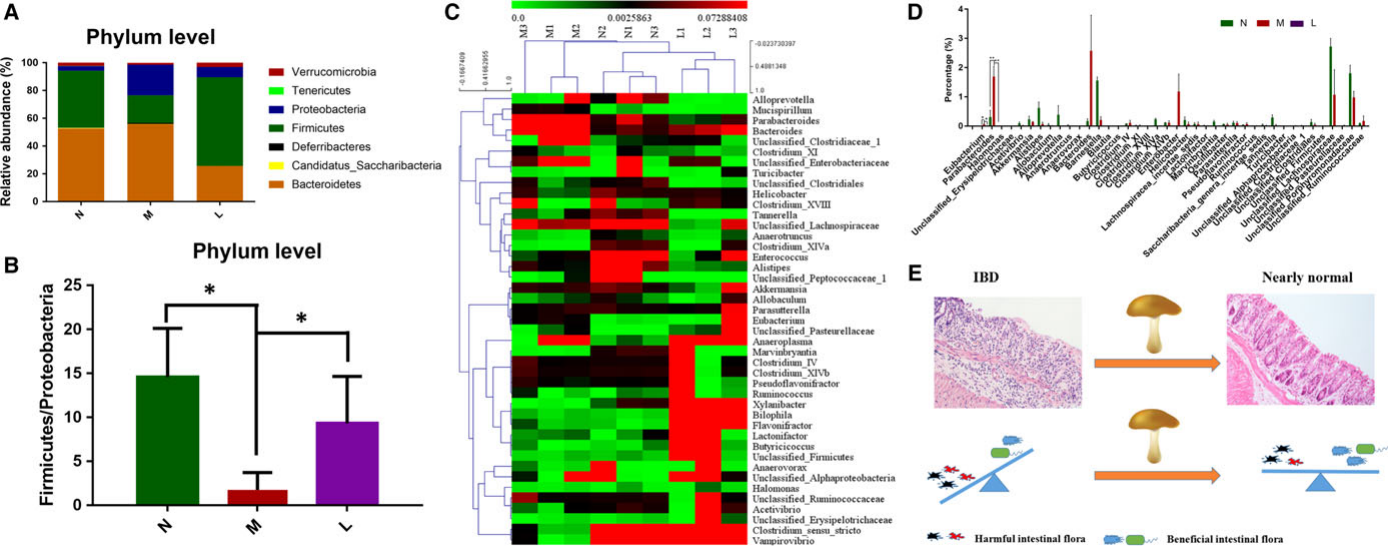
Lentinan restores intestinal bacteria microbiotal community structure in acute colitis model mice. A, Bacteroidetes, Firmicutes and Proteobacteria were the dominant bacteria at the phylum level in the mouse faecal samples. Lentinan‐treated mice were more similar to normal mice. B, Lentinan treatment recovered the Firmicutes/Proteobacteria ratio to nearly normal levels. C, Cluster analysis showed that the gut microbiota at the genus level in the lentinan‐treated mice was more similar to normal mice without acute colitis. D, Gut microbiota such as *Eubacterium* and *Parabacteroides* reduced to nearly normal levels after lentinan treatment. E, Schematic showing the role of lentinan in protecting the intestinal bacteria microbiota and mucosa

IBD is facilitated by immune hyper‐responsiveness towards certain intestinal microbiota, which is critical in stimulating mucosal immune disorders.^34^ Studies have shown that polysaccharides are capable of balancing the intestinal microbiota.^35^, ^36^ We performed 16S rRNA gene sequencing on faecal samples from normal or acute colitis model mice treated with or without lentinan to investigate changes in intestinal microbiota because of lentinan treatment. The sequencing results showed that lentinan treatment recovered the intestinal microbiotal community structure in IBD model mice. The gut microbiota on phylum levels in the lentinan‐treated mice was more similar to normal mice without colitis induction (Figure 7A). Bacteroidetes, Firmicutes and Proteobacteria were the dominant bacteria at the phylum level in the mice faecal samples. IBD model mice exhibited decreased levels of Firmicutes/Proteobacteria ratio compared with the normal mice, while lentinan treatment recovered the Firmicutes/Proteobacteria ratio to nearly normal levels (Figure 7B). This finding is consistent with a previous study that reported a decrease of Firmicutes and an increase of Proteobacteria in IBD patients.^37^, ^38^ Cluster analysis showed that the gut microbiota at the genus level in the lentinan‐treated mice was more similar to normal mice without colitis induction (Figure 7C). Specifically, gut microbiota such as *Eubacterium* and *Parabacteroides* were reduced to nearly normal levels after lentinan treatment (Figure 7D). The diagram in Figure 7E suggested that lentinan rebalances the intestinal microbiota in mice or protects the good bacteria and intestinal mucosa from harmful bacteria or other agents.

## 4 DISCUSSION

IBD is a severe disease that results in persistent patient discomfort and poor quality of life. Most of the drugs that are commonly used to treat IBD are associated with adverse effects.^39^, ^40^ Therefore, there is an urgent need to find new drugs that effectively treat IBD without severe adverse effects.

Recently, edible ginger‐derived nanoparticles were found to be effective in the prevention and treatment of IBD and CAC.^41^, ^42^ The immunomodulatory, anti‐inflammatory and anti‐carcinogenic effects of glucans, which are abundantly found in edible mushrooms, have been reported in numerous studies. These polysaccharides show high efficacy and low toxicity than other therapeutic agents in clinical use.^43^, ^44^ Lentinan is an anti‐tumour polysaccharide from *Lentinula edodes*, which is highly effective against DSS‐induced acute colitis.^20^ In the present study, lentinan suppressed body weight loss and colon length shortening and improved histological scores in DSS‐induced acute colitis, chronic colitis and TNBS‐induced model mice. High‐dose lentinan treatment provided better effects than the positive control drug SASP. Both the dose of lentinan and SASP used in this study were converted from the maximum clinical dose of the two drugs, and thus the treatment effects are comparable. These findings suggest that lentinan has immense therapeutic potential for IBD patients. Lentinan has been used clinically for many years and has very few and mild adverse effects. Lentinan prevented IBD in the present study. Moreover, lentinan inhibited tumour formation, infiltration of inflammatory cells and atypical hyperplasia in AOM/DSS‐induced CAC mice model.

Studies have shown that environment, genetics and host immunity form a multi‐dimensional, highly interactive regulatory triad controlling TLR function in the intestinal mucosa. Aberrant TLR signalling owing to environmental perturbations or genetic mutations or changes in host immunity cause acute and chronic intestinal inflammation that eventually lead to IBD and CAC if not effectively treated.^34^, ^45^, ^46^ LPS is an endotoxin released by Gram‐negative bacteria, which binds and activates TLR4 in macrophages.^47^, ^48^ This results in the activation of the MyD88 and TRIF intracellular signalling pathways, release of calcium ions and translocation of NF‐κB, thereby inducing the expression of pro‐inflammatory cytokines that can lead to IBD and CAC, if persistent.^49^, ^50^

The effects of lentinan on inflammation have been previously studied. Ahn found that lentinan attenuates AIM2 and non‐canonical inflammation while increasing pro‐inflammatory cytokines via TLR.^51^ In the current study, we found that lentinan down‐regulated LPS‐induced TLR4‐mediated NF‐κB signalling pathway and the expression of various inflammatory cytokines, which are key factors in leucocyte extravasation, colitis and CAC.^52^ For example, it reduced the expression of IL‐13 and CD30L, which are two key factors in IBD and CAC.^53^, ^54^ We suppose that the inflammation‐modulating properties of lentinan may vary depending on cell type and the inflammatory microenvironment. The glycan chains of lentinan may interfere with the binding of TLR4 to LPS and inhibit TLR4 signalling and inflammation.

Intestinal bacteria microbiota constitutes a complex microecosystem that plays a crucial role in preventing IBD and CAC. Intestinal microbiotal imbalances trigger intestinal mucosal immune disorders.^55^ The findings of this study show that lentinan promotes the recovery of the intestinal bacteria microbiotal community structures in IBD mice, which further attenuates the factors that induce IBD. We found that gut microbiota such as *Eubacterium* and *Parabacteroides* increased in IBD model mice, while lentinan reduced the relative abundance. There are conflicting reports on the role of *Parabacteroides* and *Eubacterium* in IBD. The abundance of *Parabacteroides* was increased in colitis‐prone NHE3−/− mice but was decreased in colitis‐prone IL22−/− mice.^56^, ^57^ Previous studies have found decreased and increased colonization of *Eubacterium* in UC and CD patients respectively.^58^, ^59^, ^60^ This study may enrich the relevant knowledge of intestinal microbiota changes in IBD.

## 5 CONCLUSIONS

Our study proved that lentinan is effective in treating IBD and CAC in mouse models. The inhibition of the TLR4 signalling pathway and recovery of intestinal bacteria microbiotal structures may be the potential pharmacological mechanism of lentinan in mouse models of IBD and CAC. Therefore, we demonstrate that lentinan has promising therapeutic potential in IBD and CAC treatment and prevention.

## CONFLICT OF INTEREST

The authors declare that they have no competing interests.
