# Corrigendum

**DOI:** 10.1111/jcmm.17588

**Published:** 2023-01-01

The published article entitled “Therapeutic effects of lentinan on inflammatory bowel disease and colitis‐associated cancer” with original manuscript ID of JCMM‐06‐2018‐109.R1 contains an error in Figure 3D. The incorrect images of colons from untreated and lentinan pre‐treated model mice were used in the original publication. The corrected Figure 3 is shown below. The authors confirm that all the results and conclusions of this article remain unchanged. The figure legend is unchanged.

**FIGURE 3:**
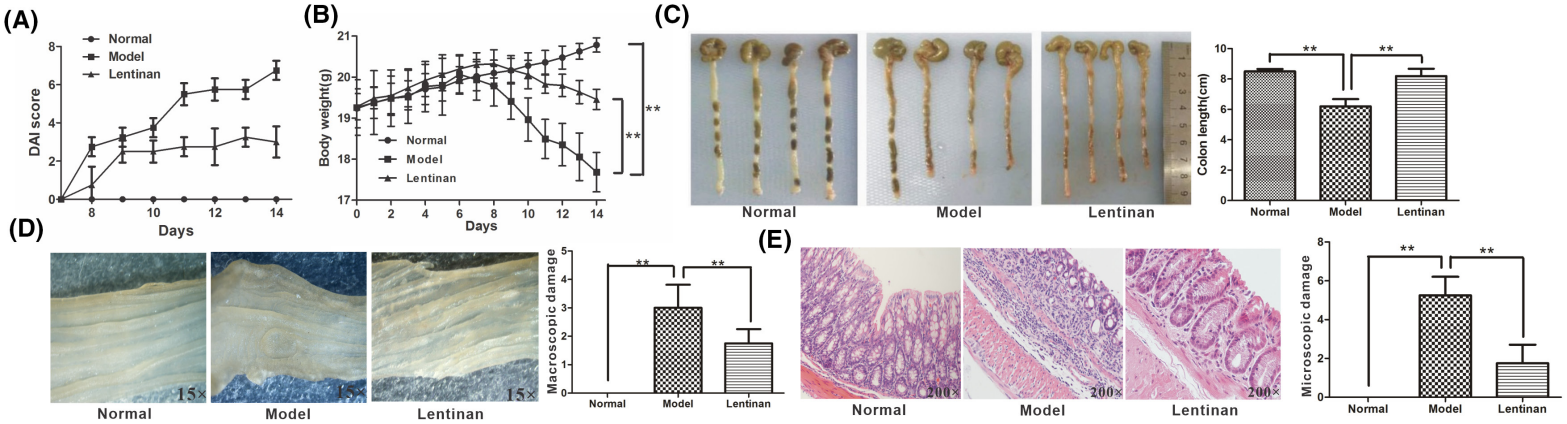
Lentinan pre‐treatment prevents DSS‐induced colitis. (A) DAIs of normal, untreated and lentinan pre‐treated colitis model mice. (B) Body weights of normal, untreated and lentinan pre‐treated colitis model mice. (C), Colon lengths of normal, untreated and lentinan pre‐treated colitis model mice. (D) Representative photographs of colons from normal, untreated and lentinan pre‐treated model mice and their macroscopic damage scores. (E) Representative photographs of H&E‐stained colon sections from normal, untreated and lentinan pre‐treated model mice and their colon histological scores.

The authors apologized for the mistakes.

