# Supplementary material for: Therapeutic effects of lentinan on inflammatory bowel disease and colitis‐associated cancer
Source: J Cell Mol Med. 2018 Nov 24;23(2):750–60. doi: 10.1111/jcmm.13897 (PMC6349230; doi:10.1111/jcmm.13897)
Supplement: Supplementary file 1 [file JCMM-23-750-s001.docx]

**Supplementary data**

1. **Supplementary Methods**

***1.1Assessment of colitis severity***

Mice were monitored for body weight, stool consistency, and bloody stool every day (for acute UC and CD) or every other day (for chronic UC) until sacrificed. Scoring for weight loss was as follows: 0 = none, 1 = 1 ~ 5%, 2 = 6 ~ 10%, 3 = 11 ~ 15% and 4 = more than 15%. In addition, mouse feces were evaluated in an observer blinded manner for consistency/diarrhea and the presence of bloody stool. Fecal pellets were given a stool consistency score of 0 = normal fecal pellet, 1 = slightly loose stool, 2 = loose stool, 3 = diarrhea and 4 = visible blood. Then we took the sum of these two scores as the disease activity index (DAI). The length of the colon, which was measured from the cecum to the anus, served as an indirect marker of the intensity of inflammation.

***1.2*** ***Assessment of macroscopic damage of colon tissues***

Colon samples fixed in 10% neutral buffered formalin were used to assess colonic damage with a stereomicroscope. For UC induced by DSS, the criteria were graded as follows: 0 = no ulcer, no inflammation; 1 = no ulcer, local hyperemia; 2 = ulceration without hyperemia; 3 = ulceration and inflammation at one site only; 4 = two or more sites of ulceration and inflammation; and 5 = ulceration extending more than 1 cm. For CD induced by TNBS, the scoring was performed according to the following parameters: adhesion (0 ~ 4; none, small parts, moderate, large parts, and severe), edema (0 ~ 1; none, exsit), necrosis (0 ~ 3; none, slight, moderate, and severe), perforation (0 ~ 1; none, exsit), ulcers and inflammation (0 ~ 4; none, local hyperemia, ulceration without hyperemia, ulceration and inflammation at one site only, two or more sites of ulceration and inflammation).

***1.3 Assessment of microscopic damage of colon tissues***

Samples were excised from the ulcerous colon, fixed in 10% neutral buffered formalin, and embedded in paraffin blocks. Slices with 4 μm sections were stained with hematoxylin and eosin (HE). The sections were examined microscopically by two pathologists in a blinded manner. The assessment should take into account of the extent of intestinal mucosal damage and inflammatory cell infiltration. For intestinal mucosal damage: 0 = no damage, 1 = discrete mucosal epithelium damage, 2 = superficial mucosal erosion, and 3 = extensive mucosal damage and extent to the deep intestinal wall. For inflammatory cells infiltration: 0 = none or small amount of inflammatory cells in the lamina propria, 1 = large or increased numbers of inflammatory cells in the lamina propria, 2 = the inflammatory cells spread to the submucosa and 3 = the whole layer has inflammatory cells infiltration.

***1.4 Western –blot detection***

The total protein for western blot analysis was extracted using 2×loading. Equal amounts of the samples were mixed with sample buffer [125 mM Tris-HCl (pH 6.8), 4% SDS, 10% 2-mercaptoethanol, 0.2% bromophenol blue, and 20% glycerol] in a 1:1 ratio, boiled for 30 min, and electrophoresed on SDS-PAGE, and membranes were incubated with a series of antibody (Table S1). Blots were developed using HRP-conjugated secondary antibodies and the membrane was exposed to a hyper film in the film cassette. For Western blot analysis of pNF-κB p65, nuclear protein was extracted using a NE-PER kit (Thermo). Other steps were performed as described above. Equal amounts of the samples were electrophoresed on SDS-PAGE, and membranes were incubated with anti- p-NF-kB p65 (Cell Signaling) and anti-Lamin B1 (Epitomics).

***1.5 Dual-Luciferase Reporter Assays***

Transfection efficiency was controlled by co-transfection of Renilla luciferase reporter plasmid pRL-TK plasmid(Beyotime Biotechnology, Haimen, China)using Lipofectamine TM 2000 (Invitrogen Carlsbad, California, CA,USA) according to the manufacturer's instructions for 6 h. Cells were further incubated with fresh medium for 24 h. After that, cells were co-treated with LPS (1μg/mL) and different concentrations of lentinan (0.5, 1 and 2mg/mL). After 24 hours of incubation, cells were lysed by 1× lysis buffer and luminescence was measured with a Luminoskan Ascent Reader System (Thermo, Waltham, Massachusetts, USA).

***1.6 Immunofluorescence detection of NF-κB-p65 complex***

RAW 264.7 macrophages were seeded in cell slides of 24-well plates at a density of 10^5^cells/well and incubated overnight. The cells were incubated for 24 hours with 1mg/mL of lentinan or the PBS control and then induced by 1µg/ml of LPS for 30 minutes. After induction, the cells were fixed in 4% paraformaldehyde for 20 min and permeabilized for 5 minutes in PBS 0.2% triton. After blocking, the cells were incubated with rabbit anti-NF-κB-p65 antibody (Affinity) and with anti-rabbit conjugated with FITC secondary antibody (EarthOx). Cells were covered with mounting medium containing 4-,6-diamidino-2-phenylindole (DAPI). Observations and acquisition were performed with a confocal microscope.

***1.7 Immunohistochemical analyses of colon tissues***

Immunohistochemical staining was performed on paraffin-embedded slides of colon sections. Sections were deparaffinized and rehydrated, and endogenous peroxidase activity was blocked with 3% H_2_O_2_. Then heat-induced antigen retrieval was performed, using 0.01 M citrate buffer (pH 6.0) for 10 min preheating and 6 min boiling in a microwave, followed by blocking with 1% BSA for 30 min. The sections were incubated with the primary antibodies (Table S2) overnight at 4℃. Subsequently, incubation with horseradish peroxidase (HRP) or Alkaline phosphatase (AP) conjugated secondary antibodies and substrate DAB addition was performed using an EnViSion Detection Kit(Fuzhou Maixin Biotech. Co. Ltd, Fujian, China) following the manufacturer's instructions. Finally, sections were counterstained with hematoxylin.

***1.8 16S rRNA sequence***

The total genomic DNA from each fecal sample (100 mg) was extracted using a QIA amp DNA stool minikit according to the manufacturer's instructions, resuspended in 100μl of TE buffer (pH 8.0), and quantified with an Eppendorf biophotometer. In order to develop phylum- and class-specific primers, between 20 and 30 16S rRNA sequences of each dominant genus of the target taxonomic group were randomly downloaded from the Ribosomal Database Project II (RDP-II) and grouped into Fasta files[[1](#_ENREF_1), [2](#_ENREF_2)].Sequences from each taxon were clustered using ClustalX[[3](#_ENREF_3)], and consensus sequences were obtained using BioEdit. The consensus sequences were aligned with the Multalin program. The alignments of these consensus sequences were visually inspected to design primers. It is known that nucleotide mismatches at the primer's 3′ termini are extended by DNA polymerases with lower efficiency than correctly matched. Therefore, primers were designed to possess the taxon-specific nucleotide(s) at the 3′end[[1](#_ENREF_1)]. Primers were assessed in silico using the tool “probe match” from the RDP-II and compared to available 16S rRNA gene sequences by using the NCBI BLAST database search program. These samples were analyzed with the Quantitative Real-time PCR (qPCR), and the Cyclethres hold (CTs ) were used to calculate the proportion of higher bacterial taxa in the feces.

1. **Supplementary figure legends**

**Figure S1. The structure of lentinan**


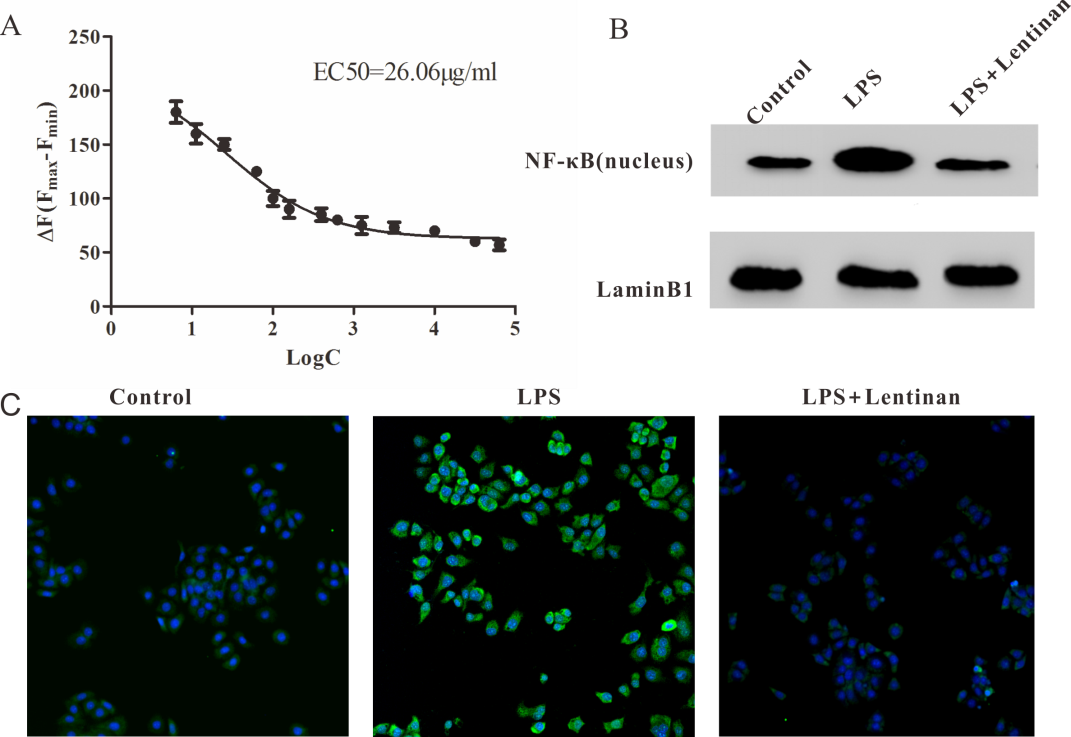


**Figure S2.** Lentinan interferes with the TLR4 signaling pathway in Caco-2 cells. A. The calcium influx signal in the Caco-2 cells decreases in a dose dependent-manner when the cells are treated with lentinan. B. Western blot analysis of the levels of the NF-κB p65 subunit in the nuclear extract from Caco-2 cells. C. Translocation of NF-κB-p65 to the nucleus, as assessed by immunofluorescence.

**
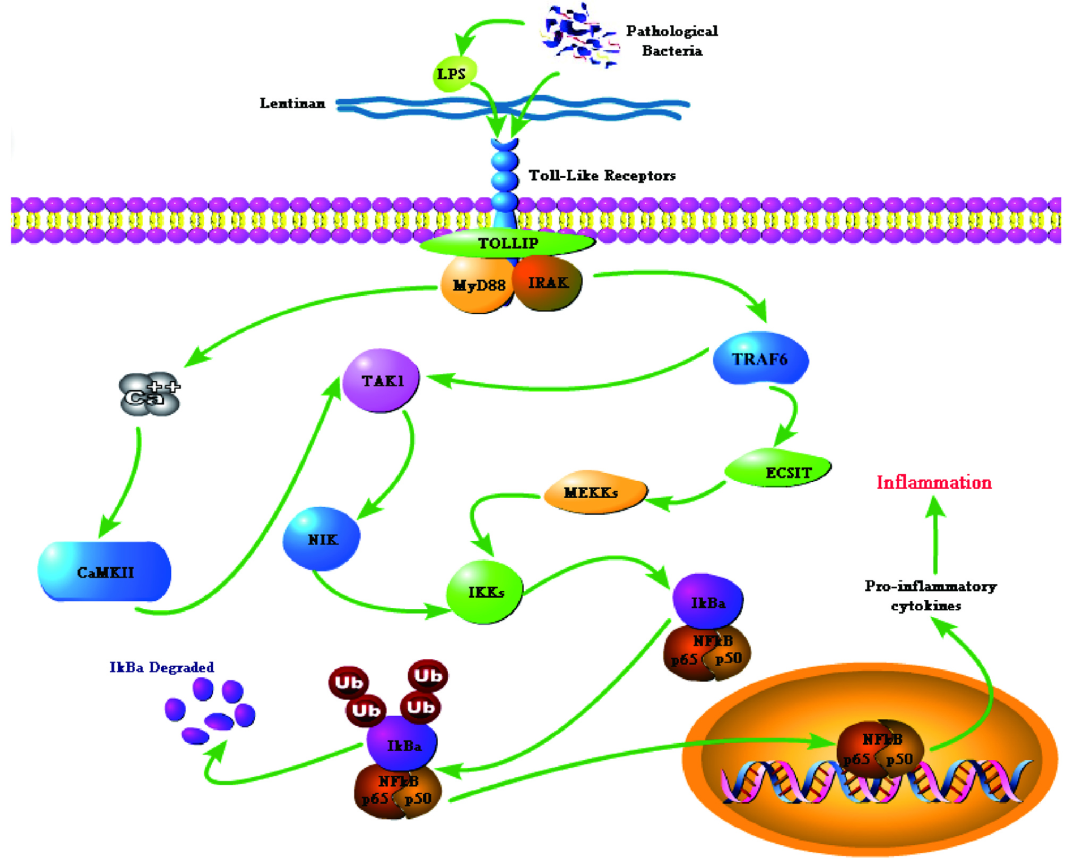
**

**Figure S3.** The schematic diagram that lentinan regulated TLR4 signalling pathway.

| **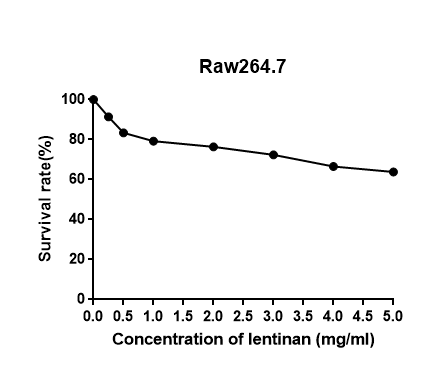** | **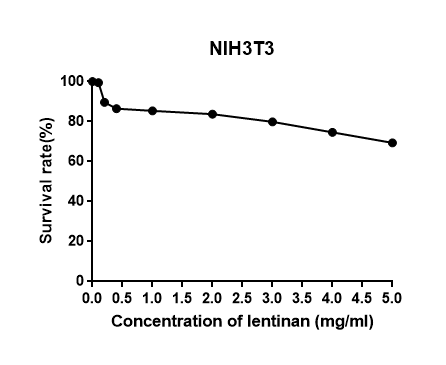** |
| --- | --- |

**Figure S4.** The cytotoxicity of Lentinan in vitro. MTT assays were used for determine the inhibition of lentinan on the survival rate of normal cell lines including RAW264.7 and NIH3T3.

**References**

1. Bacchetti De Gregoris T, Aldred N, Clare AS and Burgess JG. Improvement of phylum- and class-specific primers for real-time PCR quantification of bacterial taxa. Journal of microbiological methods. 2011; 86(3):351-356.

2. Cole JR, Chai B, Farris RJ, Wang Q, Kulam-Syed-Mohideen AS, McGarrell DM, Bandela AM, Cardenas E, Garrity GM and Tiedje JM. The ribosomal database project (RDP-II): introducing myRDP space and quality controlled public data. Nucleic acids research. 2007; 35(Database issue):D169-172.

3. Kumar M. An enhanced algorithm for multiple sequence alignment of protein sequences using genetic algorithm. EXCLI journal. 2015; 14:1232-1255.
